# Development of a patient advocate committee for a pediatric biobank

**DOI:** 10.1186/s40900-025-00834-9

**Published:** 2026-03-04

**Authors:** Frances Argento, Mackenzie Devlin, Serge Dorna, Kaitlyn Flegg, Michelle Prunier, Tim Regan, Ivana Ristevski, Lynn Tarabey, Helen Dimaras

**Affiliations:** 1 Department of Ophthalmology & Vision Sciences, The Hospital for Sick Children, 555 University Ave, Toronto, ON M5G 1X8 Canada; 2 Institute of Medical Sciences, University of Toronto, 1 King’s College Cir Suite 2374, Toronto, ON M5S 3K Canada; 3 Patient Partner, Kids Eye Biobank, Toronto, Canada; 4 Department of Ophthalmology & Vision Sciences, University of Toronto, 144 College Street, Toronto, ON M5S 3M2 Canada; 5 Division of Clinical Public Health, Dalla Lana School of Public Health, University of Toronto, 150 College Street, Toronto, ON M5T 3M7 Canada

**Keywords:** Patient engagement, Patient and public involvement, Methodology, Governance, Patient advisory committee, Biobank, Pediatric, Ophthalmology, Oncology, Ocular cancer

## Abstract

**Background:**

The Kids Eye Biobank is a pediatric ophthalmology biobank with a dedicated eye cancer collection. It centralizes data (e.g., biological samples, images, and clinical data) for use in future research. Recognizing the importance of patient engagement in research, the Kids Eye Biobank was designed with a patient-centered governance model, which includes a patient advocate committee (PAC). This study describes the development of the PAC and its outputs.

**Methods:**

Patients (defined as anyone with lived experience of pediatric eye disease) were recruited by contacting pre-existing research partners, patient advocacy networks (e.g., Canadian Retinoblastoma Research Advisory Board), and biobank participants/families. A patient engagement plan was created to guide the PAC's work; PAC members refined and prioritized goals. The PAC reviewed the Kids Eye Biobank’s governance structure and were invited to sit on additional committees. Monthly PAC meetings were held, during which members worked on implementing goals outlined in the patient engagement plan.

**Results:**

As of August 2025, there were six PAC members; five live in North America and one lives outside of North America. Two members had eye cancer as children, three were parents of children with eye cancer, and one member had dual experience. The PAC held 19 virtual meetings. PAC members ranked goals within the patient engagement plan, choosing to focus efforts on Biobank participant recruitment, PAC member mentorship, and contributing lived experience to the Kids Eye Biobank’s informed consent process. A “Participant Information Pamphlet” was reviewed and edited to recruit biobank participants. The PAC created an “Information Guide” to sensitize and train new patient partners. After reviewing the informed consent process, PAC members identified the need for and created a patient-centered tool to facilitate informed consent discussions. Additionally, three PAC members joined other governing committees to enhance the Kids Eye Biobank’s patient-oriented operations.

**Conclusions:**

PAC member commitment is demonstrated by regular meetings, project progression and involvement throughout the Kids Eye Biobank’s governance and operations. Patient partners made unique contributions from their lived experience to suggest enhancements to the Kids Eye Biobank’s communications, related to participant recruitment, PAC member mentorship, and the informed consent process.

**Supplementary information:**

The online version contains supplementary material available at 10.1186/s40900-025-00834-9.

## Abstract (pa)

**Background:**

The Kids Eye Biobank collects images, health notes, and samples from people, like blood, spit, or small pieces of tissue. These are safely stored and shared for use in future research. Partnering with patients and their family members is important to the Kids Eye Biobank because it can help improve the quality and impact of research. Therefore, a Patient Advocate Committee (PAC) was created as a way for patients to help lead the Kids Eye Biobank. This paper describes how the PAC was set up and its outcomes.

**Methods:**

PAC members were recruited by contacting previous research partners, community groups, and Kids Eye Biobank participants and family members. Once recruited, PAC members created a plan to guide their roles and activities. PAC members met monthly and together, worked through their plan.

**Results:**

As of August 2025, the PAC has six members. All members have personal experience with retinoblastoma, a childhood eye cancer. Over the course of 19 online meetings, the PAC created different print and electronic materials to help the Kids Eye Biobank recruit participants and mentor new PAC members. The PAC members also joined additional committees in the Kids Eye Biobank.

**Significance:**

The PAC shared special insights, which were primarily used to help improve the Kids Eye Biobank’s communications. This included how the Kids Eye Biobank speaks with potential participants and supports new PAC members. Overall, PAC members demonstrated their commitment by regularly attending meetings, completing multiple projects, and joining additional committees.

## Background

Biobank facilities collect and store biological specimens (e.g., tissues, saliva, blood), medical images, and annotated clinical data for future research use [1]. Through aggregating diverse data types, biobanks form comprehensive repositories that can streamline researchers’ access to resources, and empower collaborative scientific endeavors [1, 2]. Biobanks have the potential to support a wide range of research related to pediatric ophthalmology—defined as the diagnosis, treatment, and management of eye and vision conditions in children—as many childhood eye conditions are challenging to diagnose, rare, complex and have lifelong impacts [3, 4]. Moreover, biobanking may be particularly advantageous for pediatric ocular oncology research (a subset of pediatric ophthalmology), which deals with rare pediatric eye cancers (R-PECs). R-PECs are a group of diverse neoplasms—an abnormal or excessive growth of cells—that affect the eye and/or its surrounding structures and threaten vision, eye loss, quality of life and patient survival [4, 5]. Due to their rarity, heterogeneity, and geographically dispersed incidence, R-PECs pose unique research challenges [6]. This has confined previous research to small sample sizes and limited the robustness of study data [6]. The centralized nature of biobanking offers a strategic approach to overcome these limitations and further catalyze novel genomic, translational, and epidemiological studies [7].

Biobanks that aim to collect data over long periods of time can provide deeper insights into conditions that manifest in childhood [8]. This longitudinal approach may be advantageous for studying ophthalmic diseases such as R-PECs, many of which are associated with congenital disorders or different phases of development [4]. For example, approximately 40% of retinoblastoma cases—cancer of the infant retina—are heritable [9]. These individuals may pass on genetic traits to their offspring that pre-dispose them to cancer development [9]. Additionally, pediatric uveal melanoma, referring to cancer of the middle layer of the eye, including the iris, ciliary body, or choroid, often occurs in individuals over the age of 10, and it has been suggested that puberty plays a role in the formation of this cancer [10].

Patient engagement serves as a foundation for meaningful and impactful research practice [11]. It is defined as the “active and meaningful partnership with patients throughout all stages of the research process.” [11]. Benefits include increased research relevance, enhanced quality, improved recruitment efforts, and streamlined knowledge translation between researchers and patients [12, 13]. Meaningful patient engagement recognizes that patients (defined as anyone with lived experience of disease, including informal care givers) are experts in their lived experience [11]. When patients partner in research, they transition from a traditional role of passive research participant to active partner [11]. Engagement can occur in different forms and at different levels, which the International Association for Public Participation (IAP2) describes as informing, consulting, involving, collaborating, and empowering [14]. While all forms of engagement are important, the deepest levels are considered to be the most valuable and have the greatest positive impact on research [14].

The importance of patient engagement in research has been demonstrated in the rare eye cancer and pediatric ophthalmology community [15, 16]. A growing number of adult-focused biobanks are also adopting patient engagement approaches [17]. However, comprehensive documentation of patient engagement methods in biobanking remains rare [17–21]. This evidence base is even more sparse in the context of pediatric biobanking, which may require tailored patient engagement approaches due to their unique populations and rare disease types [8].

Recognizing the importance of patient engagement in biobanking, the Kids Eye Biobank (a pediatric ophthalmology biobank with a dedicated R-PEC collection), was designed to have a patient-oriented governance structure, including a patient advocate committee (PAC) [22]. Herein, we describe the methods undertaken to establish the PAC and its early outputs.

## Methods

### Aims

The primary aim of this project was to establish and develop a PAC in a pediatric ophthalmology biobank. This paper describes the methods of patient engagement used, the PAC's operations, activities, and early outputs.

### Study setting

The Kids Eye Biobank is located at the Hospital for Sick Children (SickKids) in Toronto, Canada. The Kids Eye Biobank is a pediatric ophthalmology biobank that collects data from individuals with various eye and vision diseases. To our knowledge, the Kids Eye Biobank is the first pediatric ophthalmology biobank with a dedicated R-PEC collection. The Kids Eye Biobank has a detailed governance structure which was designed to facilitate patient engagement through multiple committees [22]. For this study we decided to establish a PAC focusing first on individuals with lived experience of R-PECs. This research will serve as proof of principle, with the intention of being scaled up to encompass patient engagement in biobanking for other ophthalmic conditions.

### PAC member recruitment

Patients with lived experience of a R-PEC were eligible to become members of the PAC. In the first phase of PAC recruitment, the Kids Eye Biobank team utilized pre-existing research relationships and contacted relevant advocacy groups (e.g., the Canadian Retinoblastoma Research Advisory Board (CRRAB)). After the PAC was established, members reviewed recruitment strategies and provided feedback on how to expand recruitment efforts (see Results).

### Committee policies

#### Terms of Reference

A preliminary Terms of Reference (ToR) was drafted by the Kids Eye Biobank team. The ToR outlined the PAC's mandate, member responsibilities, membership and term, and meeting frequency. The ToR was circulated to PAC members before the first meeting. PAC members were invited to review the ToR’s contents. In the first PAC meeting the ToR was discussed and refined.

#### Compensation policy

The Kids Eye Biobank compensation policy was created to establish clear expectations for patient partner compensation. The compensation policy was informed by guidelines set out by the Canadian Institute of Health Research (CIHR), AbSPORU Patient Engagement Team, and Strategy for Patient Oriented Research [23, 24].

### Patient engagement plan

A preliminary patient engagement plan (PEP) was drafted by the Kids Eye Biobank team and circulated to PAC members in advance of the first meeting. The PEP consisted of overarching goals to guide the PAC's work, and measurable tasks for each goal. The purpose of the PEP was to serve as a guiding framework for the PAC's operations, and guide meeting focus. PAC members revised the PEP during the first PAC meeting, and over the course of its operations when deemed necessary by PAC members.

### PAC meetings

#### PAC facilitator

The PAC and its activities were guided by author FA, who served as a meeting facilitator and liaison between the Kids Eye Biobank team and PAC.

#### Meeting organization

PAC members collectively discussed and agreed on the regular meeting date, time and frequency. Meetings were scheduled up to six months in advance. Microsoft Outlook and WhatsApp were used to communicate between the meeting facilitator, PAC members and Kids Eye Biobank team. PAC meetings were scheduled monthly on Tuesday evenings at 8:00 PM Eastern Standard Time (EST). Members decided that this time best accommodated everyone’s schedules; one PAC member lived in a different time zone, many PAC members are parents with variable weeknight availability. A meeting agenda was created in advance of every PAC meeting. The week prior to each PAC meeting, a meeting reminder and any necessary documentation was sent out to PAC members. Brief notes were taken throughout the meeting. All meetings were recorded and stored in the Kids Eye Biobank’s records. Meeting recordings were shared with PAC members if they were unable to attend a meeting or wished to review meeting content. The day after each meeting, a follow-up email was sent to members highlighting topics covered, next tasks, and future expectations. Records for each meeting were maintained; details included the date of each meeting, agenda, and members in attendance.

#### Meeting facilitation

Monthly meetings were utilized as guided working sessions to address goals outlined in the PEP. This included discussing the Kids Eye Biobank (e.g., current operations, research directions), working on projects (e.g., collaboratively providing feedback, reviewing progress), and assigning tasks. Between meetings, PAC members were asked to review materials and independently work on projects. Ad hoc project specific meetings were held between monthly meetings. Members from the Kids Eye Biobank team were occasionally invited to join meetings for consultation on different projects or discussions of the Kids Eye Biobank.

#### Human centered design

A Human Centered Design (HCD) approach was utilized by the PAC for working towards certain goals within the PEP. HCD is a user-centered problem-solving method which operates through five phases: (i) problem definition, (ii) solution ideation, (iii) rapid prototyping, (iv) refinement, and (v) evaluation [25].

### Evaluation

The Public and Patient Engagement Evaluation Tool (PPEET) is a validated survey that aims to capture the quality of patient engagement in health research [26]. PAC members were asked to fill out the PPEET after monthly meetings. The PPEET was distributed to PAC members using the Research and Electronic Data Capture (REDCap) system. The results of the PPEET will be reported on in a subsequent manuscript.

## Results

### PAC membership

Six patients were recruited to be PAC members. Five PAC members live in North America and one lives outside of North America. All PAC members had lived experience of retinoblastoma. Two PAC members had retinoblastoma as a child, three members are parents of children with retinoblastoma, and one partner has dual lived experience. Two PAC members are male and four are female. PAC members had a wide variety of professional skills and expertise, including graphic design, software engineering, clinical research, social work, communications, and education. In addition to the six official PAC members, there were three *ad-hoc* committee members including the Kids Eye Biobank Director, Manager, and Facilitator.

### Meetings

Between September 2023 and August 2025, 19 PAC meetings were held. All meetings were 60 minutes in duration. The overall attendance rate of meetings by PAC members was 71%. Additionally, an in-person social was held in April of 2024, that was 1 hour in length. Ten additional 30-minute ad-hoc project specific meetings were held between the Facilitator and a PAC member.

#### First PAC meeting

The first PAC meeting held in September 2023 served to introduce and better acquaint members with each other. This meeting was also used to help educate PAC members on the Kids Eye Biobank and address knowledge gaps. Ad hoc members created presentations that explained the Kids Eye Biobank and the proposed purpose of the PAC. This meeting was important for setting expectations between PAC members and the Kids Eye Biobank team. One of the first requests from PAC members was to get visuals of the biobank to better understand the physical storage system. In response, a video of the Kids Eye Biobank storage facility and specimen processing was created.

### Patient engagement plan outputs

During the first PAC meeting members revised the PEP, defined respective goals and their tasks, as well as the estimated time commitment. There are eight goals in the PEP [see Additional File 1]. The PAC refined each goal and collectively described the order in which they were to be addressed. The top five goals are described in detail below.

#### Goal 1: Establish and operate an R-PEC diverse PAC

The first goal within the PEP was to “Establish and Operate an R-PEC Diverse PAC”. Deliverables within this goal included: (i) establishing a regular PAC meeting schedule, (ii) developing a PAC member recruitment plan, (iii) creating outreach and promotional materials and (iv) recruiting patient advocates. From the Kids Eye Biobank’s initial efforts, five PAC members were recruited. The PAC performed a second round of recruitment, contacting 11 cancer-related organizations, 5 Facebook groups, and 30 Kids Eye Biobank families directly to recruit patient partners. The PAC also hosted an information booth at the 2024 CRRAB Annual Symposium. One additional member was recruited in March 2024, totaling six PAC members.

#### Goal 2: Recruit and mentor new patient partners

**Fig. 1:**
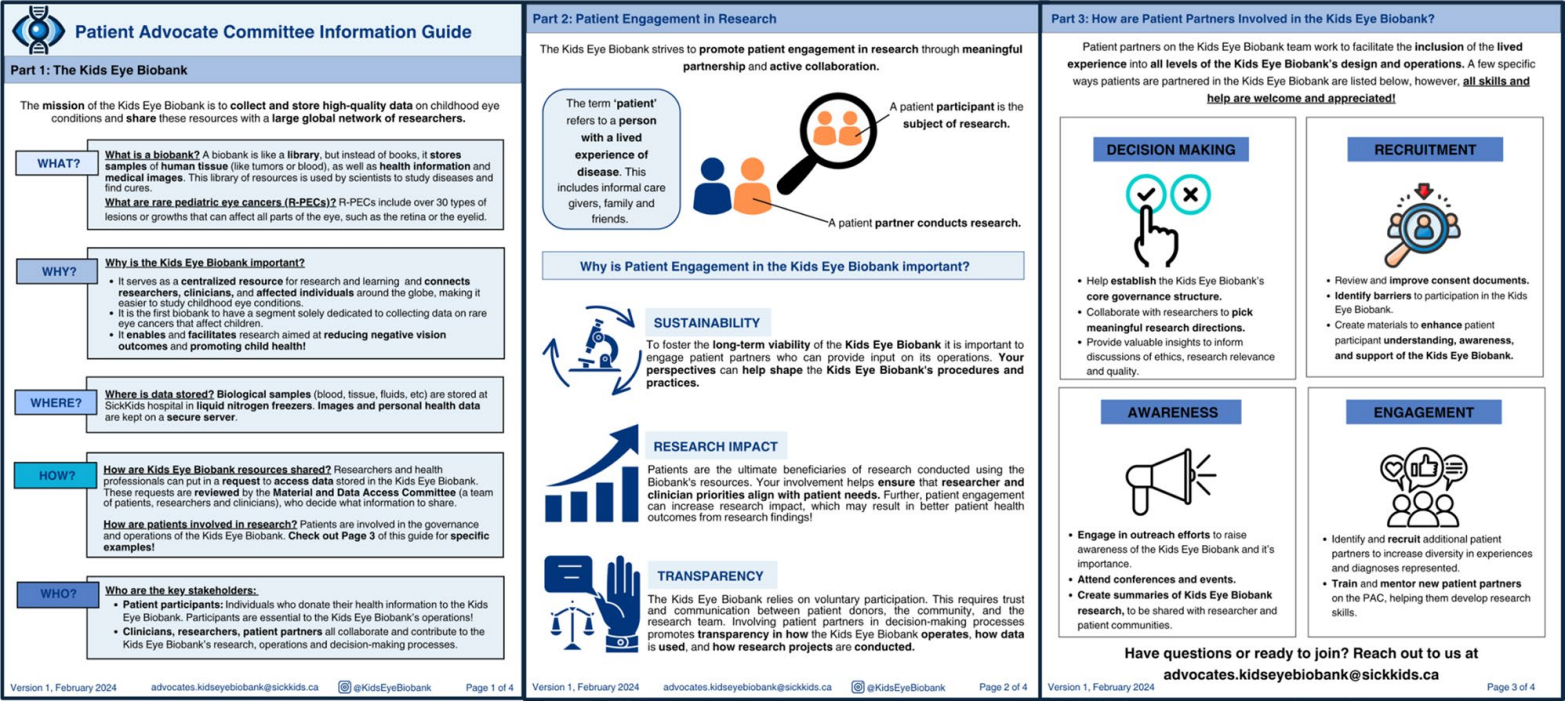
PAC Information Guide. The PAC Information Guide was created to enhance PAC member recruitment and mentor new patient partners. The PAC Information Guide has three parts. Part 1, ‘The Kids Eye Biobank’ addresses frequently asked questions pertaining to biobanking. Part 2, ‘Patient Engagement in Research’ defines patient partnership and why it is important in research. Part 3, ‘How are Patient Partners Involved in the Kids Eye Biobank’ provides examples of patient engagement in biobanking

The PAC collaboratively developed a “PAC Information Guide” [Fig. 1] for the Kids Eye Biobank to facilitate recruitment and mentorship of new PAC members. Through structured discussions, PAC members drew from their lived experiences to identify key information that was the most relevant for prospective members. PAC members reflected on questions they initially had about the Kids Eye Biobank, and used this to decide what information should be included in the PAC Information Guide. Further, PAC members advocated for minimizing text and using simple graphics to explain complex biobanking concepts. Canva was used to design the PAC Information Guide [27].

#### Goal 3: Enhance patient oriented operations throughout the Kids Eye Biobank.

The Kids Eye Biobank governance structure was designed to facilitate patient engagement by embedding patient partners across its committees. One PAC member joined the External Oversight Committee (EOC) which provides financial, ethical and legal oversight to the Kids Eye Biobank. Two PAC members joined the Material and Data Access Committee (MDAC), which reviews research requests for use of the Kids Eye Biobank’s resources and makes decisions on how data is shared.

#### Goal 4: Promote awareness of the Kids Eye Biobank among potential and current patient families.

**Fig. 2:**
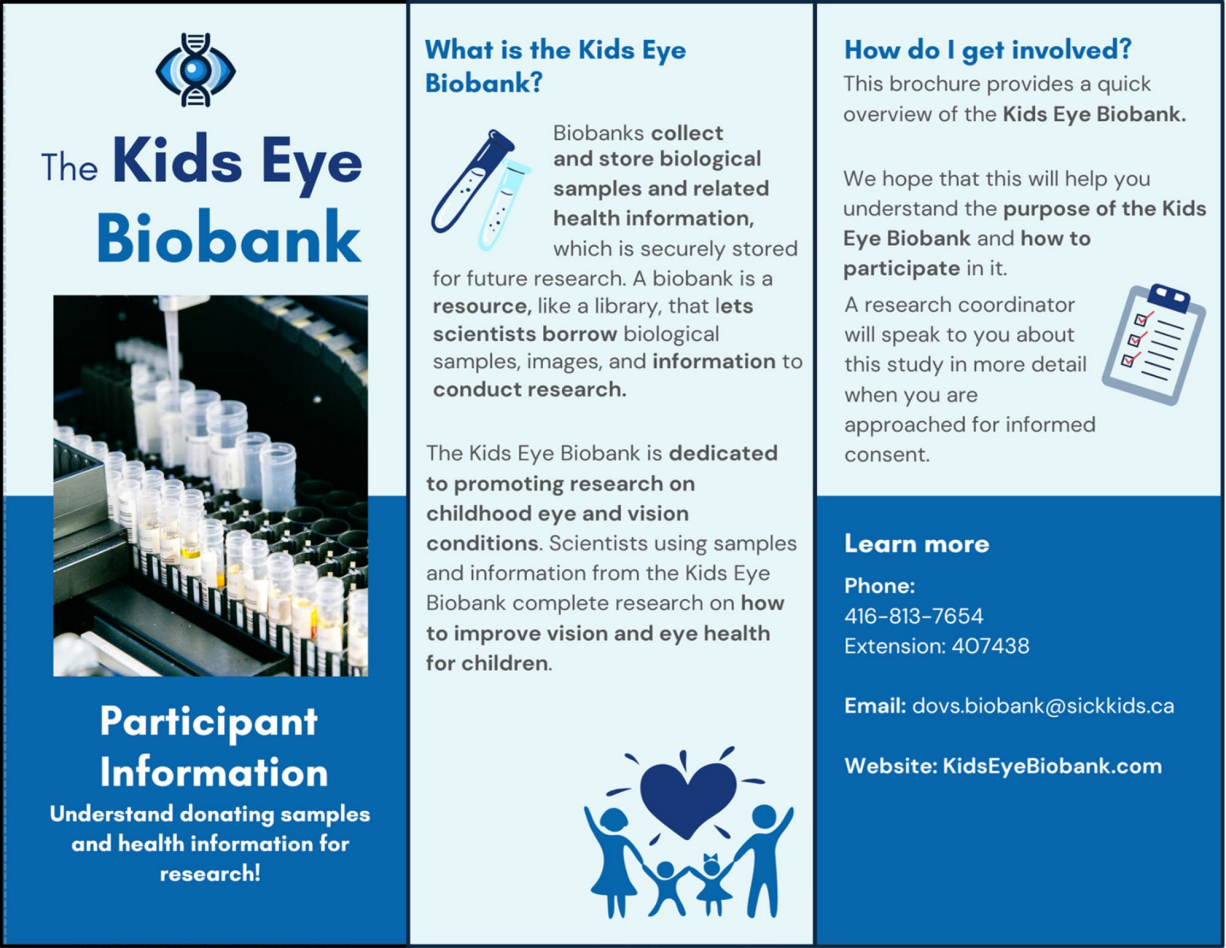
Participant Information Pamphlet. The PAC reviewed and refined a pamphlet aimed at promoting the Kids Eye Biobank and participant recruitment

The PAC designed a Kids Eye Biobank Participant Information Pamphlet [Fig. 2] to enhance awareness among potential ophthalmology patients. Members utilized their lived experience to refine pamphlet language, clarify definitions, and enhance the design of the pamphlet. The Participant Information Pamphlet is available at SickKids in the Department of Ophthalmology and Vision Sciences eye clinic, and is distributed to any interested patients.

#### Goal 5: Contribute patient expertise into the Kids Eye Biobank’s informed consent process.

**Fig. 3:**
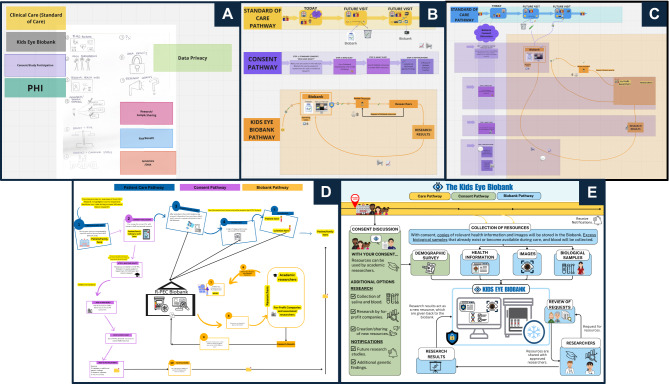
Development of the CHOICE Tool. Using a human centered design approach the PAC: (**a**) reviewed the Biobank consent form and identified important concepts, (**b**) sketched out pathways for a patient’s typical standard of care, the Kids Eye Biobank, and biobank consent decisions, (**c**) connected the pathways to each other and their interactions, (**d**) drafted an outline of the CHOICE Tool, and (**e**) created a high fidelity prototype of the CHOICE Tool in Canva

PAC members reviewed the Kids Eye Biobank’s informed consent process. Upon review, PAC members agreed that the consent form and informed consent discussions could be enhanced by patient input. Applying the HCD approach described Human Centered Design, PAC members discussed important concepts within consent materials and explored the use of graphics to aid participant understanding. The PAC created the Clear, Honest, Open, Informed Consent Experience (CHOICE) Tool; a patient-informed resource aimed at improving participant understanding during the consent process [Fig. 3]. All PAC members collaborated in the planning of this tool. The designs were led by one PAC member who has graphic design experience, using Canva [27] and Adobe Illustrator.

## Advocacy and dissemination materials

### Patient engagement roundtable discussion

PAC members aided in the design and execution of a roundtable discussion, held at the International Society for Biological and Environmental Repositories (ISBER) 2025 Annual Meeting and Exhibits. One PAC member attended and co-led the roundtable in person. Two PAC members attended virtually.

### Other advocacy

PAC members have supported the Kids Eye Biobank in multiple ways. First, PAC members attended multiple research conferences advocating for the PAC and Kids Eye Biobank. All PAC members attended the 2024 CRRAB Annual Symposium; one patient partner gave a presentation on the PAC at this symposium. PAC members also advocated for Kids Eye Biobank merchandise, to demonstrate their support for the Kids Eye Biobank in their day-to-day life. Kids Eye Biobank t-shirts and stickers were designed for all PAC members.

## Key messages

While we have not yet evaluated PAC members’ experiences, patient partners provided informal reflections of their experience working with the Kids Eye Biobank.

“I enjoyed attending the meetings and felt they were very much worth my time since we made progress and completed tasks that I felt would benefit the R-PEC community”.

“This project was a great opportunity for me to be creative and contribute to something important. I was happy and felt good about what we were building, I felt we were a team and that gave me a lot of joy”.

## Discussion

This study reports on the methods undertaken to engage patients in a pediatric biobank through a PAC. Monthly meetings were used as dedicated working sessions. Project specific meetings and independent working sessions were used for additional project revision. This meeting structure reflects similar strategies reported in youth engagement literature for other research fields [28]. PAC activities yielded several notable outcomes, including collaborative goal setting with patients and the integration of patient insights into biobank communications (e.g., PAC Information Guide, Kids Eye Biobank Participant Information Pamphlet). Notably, PAC members provided unique insights on biobank consent and communication materials, ultimately creating the novel CHOICE Tool to assist with biobanking informed consent.

Patient engagement in research is widely recognized to benefit research quality and outcomes [29, 30]. There are multiple patient engagement in research frameworks, including the CIHR's Strategy for Patient-Oriented Research [31], Patient-Centered Outcomes Research Institute (PCORI) Engagement Rubric [32], IAP2 Spectrum of Public Participation [14], National Institute for Health and Care Research INVOVLE framework [33], and others [34]. Their adoption may depend on the needs of each research project and the population being engaged [28, 34]. With respect to R-PECs, the CRRAB has also outlined a framework for patient engagement in the retinoblastoma community [35]. Its success is reflected in downstream projects, including collaborative priority setting for retinoblastoma research [16], the development of a Retinoblastoma Journey Map [15], and improved knowledge translation activities [35]. While patient engagement is reported in other precision oncology networks in Canada [36] there is limited available evidence on patient engagement for other R-PECs.

**Fig. 4:**
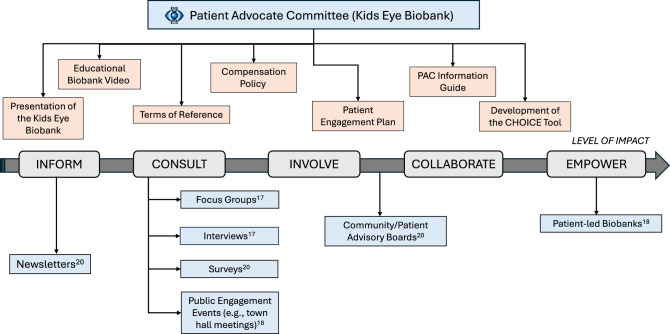
Reported examples of patient engagement in biobanking along the IAP2 Spectrum of Public Participation. Reported methods of patient engagement in biobanking (blue boxes; superscript numbers refer to relevant references that report specific examples of engagement methods) and Kids Eye Biobank Patient Advocate Committee (PAC) projects (orange boxes) are mapped across the IAP2 Spectrum of Public Participation (grey). Our findings suggest that the PAC serves as a vehicle to sustain engagement at all levels of the spectrum

It is widely recognized in the biobanking community that it is valuable to involve patients in biobank activities [17, 19]. While there are general frameworks for reporting patient involvement in research, such as the GRIPP2 Reporting checklist (Guidance for Reporting Involvement of Patients and the Public) [37], there is insufficient documentation of how patient engagement in biobanking is conducted. Further, the available literature overwhelmingly reports on biobank community engagement efforts entailing community forums, educational workshops and focus groups [17, 38, 39]. These engagement approaches tend to be initiated by researchers and may be placed at the lower end of the IAP2 Spectrum, as they often focus on *informing* and *consulting* with patients. An exception to this is the small number of patient-led biobanks, primarily in Europe, in which patient organizations have created and govern their own biobanks to expedite research on their conditions [40]. Through the PAC we achieved various levels of engagement that range across the IAP2 spectrum over the past 18 months [Fig. 4].

Many biobanks have reported using patient or community advisory committees to engage the public [20, 41–43]. These studies often lack specific details on how to approach and uphold partnership, and methods taken to achieve deeper levels of engagement (e.g*., involve, empower*). It should also be noted that there is minimal evidence (excluding testimonials) to assess the success of these engagement approaches. This challenge is not exclusive to biobanking, however, over the past decade there has been a significant increase in the number of tools and frameworks that can be used to evaluate patient engagement in research [26, 34, 44]. Recognizing this, PAC members were asked to fill out the PPEET after every PAC meeting.

Our work developing the Kids Eye Biobank PAC highlighted the dynamic nature of patient engagement; the level of patient engagement achieved did not move linearly and is not proportional to time and investment [Fig. 4]. Early meetings with the PAC were focused on relationship building, setting expectations between the PAC and Kids Eye Biobank team (bidirectional) and educating PAC members on biobanking. Educating PAC members and researchers on different aspects of biobanking and patient engagement was important for all projects, not solely at the beginning. It was important for the Kids Eye Biobank team to check in with PAC members and continually ask what resources patient partners needed. Researchers need to be aware of when power and knowledge differences limit barriers to engagement. Empowering both patient partners in their knowledge and skills can promote collaborative work.

Key messages from PAC members suggest that they enjoy their involvement in the Kids Eye Biobank and believe their efforts are valuable to others in the R-PEC community. PAC members further demonstrated their commitment to the Kids Eye Biobank by consistently attending monthly meetings and showing up prepared to discuss materials. Patient partners were also interested in the Kids Eye Biobank beyond the PAC; three patient partners joined additional governing committees, expressing a desire to partner in decision-making and biobank operations. Member commitment and regular meeting attendance may suggest sustainable patient engagement efforts to date. This is important for biobanks which have long-term operating plans and required sustained investment from stakeholders [45].

There were a few operational challenges encountered by the PAC. First, the PAC was biased in recruiting individuals with retinoblastoma due to pre-existing research relationships. There are many active patient engagement efforts within the Canadian retinoblastoma community that are needed for other R-PECs. Accordingly, the PAC had difficulty recruiting individuals with R-PECs other than retinoblastoma. Diverse recruitment of patient partners is a challenge echoed in other patient engagement literature [46]. Individuals with other R-PECs may have different research and/or biobanking priorities that were missed. However, it is not possible to determine what impact this had on the PAC. The PAC also faced logistical hurdles, including coordinating schedules for PAC members. This study also has a few limitations. The PAC speculated what levels of engagement they believe were achieved through their efforts. However, the formal evaluation of engagement is still underway and we are not able to decisively conclude this until the PPEET analysis is complete.

## Conclusions

This study provides concrete steps for establishing a PAC in a pediatric ophthalmology biobank. The PAC used multiple patient engagement approaches in an attempt to move beyond traditional informative engagement and achieve deeper levels of partnership. Overall, PAC members identified multiple patient priorities that the Kids Eye Biobank was able to work towards addressing. The methods outlined in this paper will be scaled up to recruit additional patient partners with lived experience of other ophthalmic additions. Additionally, new approaches to PAC member recruitment aimed at promoting inclusion will be discussed with PAC members and executed. An evaluation of patient engagement in the Kids Eye Biobank is planned, using data obtained from the PPEET. These survey responses will be analyzed to understand the development and impact of patient engagement on stakeholders.

## Electronic supplementary material

Below is the link to the electronic supplementary material.

Supplementary Material 1

## Data Availability

Not applicable.

## Abbreviations

CHOICE Tool: Clear Honest Open Informed Consent Experience Tool
CIHR: Canadian Institute of Health Research
CRRAB: Canadian Retinoblastoma Research Advisory Board
EOC: External Oversight Committee
GRIPP2: Guidance for Reporting Involvement of Patients and the Public
HCD: Human Centered Design
IAP2: International Association for Public Participation
ISBER: International Society for Biological and Environmental Repositories
MDAC: Material and Data Access Committee
PAC: Patient Advocate Committee
PEP: Patient Engagement Plant
PPEET: Public and Patient Engagement Evaluation Tool
REDCap: Research and Electronic Data Capture
R-PECs: Rare Pediatric Eye Cancers
ToR: Terms of Reference
SickKids: The Hospital for Sick Children
